# Effect of Magnetic Nanoparticles on Tobacco BY-2 Cell Suspension Culture 

**DOI:** 10.3390/ijerph10010047

**Published:** 2012-12-20

**Authors:** Olga Krystofova, Jiri Sochor, Ondrej Zitka, Petr Babula, Vit Kudrle, Vojtech Adam, Rene Kizek

**Affiliations:** 1 Department of Chemistry and Biochemistry, Faculty of Agronomy, Mendel University in Brno, Zemedelska 1, CZ-613 00 Brno, Czech Republic; E-Mails: olga.krystofova@seznam.cz (O.K.); sochor.jirik@seznam.cz (J.S.); zitkao@seznam.cz (O.Z.); vojtech.adam@mendelu.cz (V.A.); 2 Karel Englis College, Sujanovo nam. 356/1, CZ-602 00, Brno, Czech Republic; 3 Central European Institute of Technology, Brno University of Technology, Technicka 3058/10, CZ-616 00 Brno, Czech Republic; E-Mail: petr-babula@email.cz; 4 Department of Veterinary Ecology and Environmental Protection, Faculty of Veterinary Hygiene and Ecology, University of Veterinary and Pharmaceutical Sciences, Palackeho 1-3, CZ-612 42 Brno, Czech Republic; 5 Department of Natural Drugs, Faculty of Pharmacy, University of Veterinary and Pharmaceutical Sciences, Palackeho 1-3, CZ-612 42 Brno, Czech Republic; 6 Department of Physical Electronics, Faculty of Science, Masaryk University, Kotlarska 2, CZ-611 37 Brno, Czech Republic; E-Mail: kudrle@sci.muni.cz

**Keywords:** nanoparticles, plant cell, thiol compounds, glutathione, phytochelatin

## Abstract

Nanomaterials are structures whose exceptionality is based on their large surface, which is closely connected with reactivity and modification possibilities. Due to these properties nanomaterials are used in textile industry (antibacterial textiles with silver nanoparticles), electronics (high-resolution imaging, logical circuits on the molecular level) and medicine. Medicine represents one of the most important fields of application of nanomaterials. They are investigated in connection with targeted therapy (infectious diseases, malignant diseases) or imaging (contrast agents). Nanomaterials including nanoparticles have a great application potential in the targeted transport of pharmaceuticals. However, there are some negative properties of nanoparticles, which must be carefully solved, as hydrophobic properties leading to instability in aqueous environment, and especially their possible toxicity. Data about toxicity of nanomaterials are still scarce. Due to this fact, in this work we focused on studying of the effect of magnetic nanoparticles (NPs) and modified magnetic nanoparticles (MNPs) on tobacco BY-2 plant cell suspension culture. We aimed at examining the effect of NPs and MNPs on growth, proteosynthesis—total protein content, thiols—reduced (GSH) and oxidized (GSSG) glutathione, phytochelatins PC2-5, glutathione S-transferase (GST) activity and antioxidant activity of BY-2 cells. Whereas the effect of NPs and MNPs on growth of cell suspension culture was only moderate, significant changes were detected in all other biochemical parameters. Significant changes in protein content, phytochelatins levels and GST activity were observed in BY-2 cells treated with MNPs nanoparticles treatment. Changes were also clearly evident in the case of application of NPs. Our results demonstrate the ability of MNPs to negatively affect metabolism and induce biosynthesis of protective compounds in a plant cell model represented by BY-2 cell suspension culture. The obtained results are discussed, especially in connection with already published data. Possible mechanisms of NPs’ and MNPs’ toxicity are introduced.

## 1. Introduction

Nanomaterials are considered to be one of the most important inventions of modern science [1]. Their exceptionality is based on their constant physical properties, which are strictly dependent on their size, which varies from 1 to 100 nm. In addition, the interesting range of their properties is due to the large surface area that may be modified or functionalised for different biological applications [2]. These modifications can lead to higher water solubility or some targeting. Nanomaterials, especially nanoparticles, can be linked with different biologically active molecules, which can exactly direct them to specific sites within biomolecules including proteins and nucleic acids, sub-cellular, cellular, tissue and body structures [3,4]. Due to above mentioned properties, nanoparticles have found a wide range of applications, especially in industry (textile industry—silver nanomaterials with antibacterial properties) [5,6,7,8,9], electronics (high resolution imaging, logical circuits on the molecular levels) [10,11,12,13], agriculture (wastewater treatment), cosmetics (TiO_2_ as UV protective agent) and medicine [14,15]. The possibility of nanoparticle targeting enables their application in imaging methods, and in the transport of active compounds in the treatment of different threats, especially malignant ones [3,16,17,18,19,20]. However, nanomaterials represent possible dangers, both medically and environmentally. Their catalytic properties, which are based on the large bioactive surface, are almost unknown. In addition, nanomaterials can pass through cell membranes, however, their interactions with biomolecules and cell structures still remain almost unknown. From this point of view, attention has been paid to fullerenes (C_60_), carbon nanotubes and nanofibers, quantum dots, different metal oxide-based nanoparticles (TiO_2_, ZnO, Fe_2_O_3_, Fe_3_O_4_, CuO, CeO_2_, Al_2_O_3_), and metal-based nanoparticles (Au, Ag, Cu, Co, Ni, *etc.*) [21]. The second view on the risk of nanomaterials usage represents their possible accumulation in the environment with subsequent entry into the food chain, which is closely connected with their accumulation in organisms [22,23]. 

Plants represent an important trophic level [24]. However, there are only limited studies focused on the effect of nanomaterials on plants. The first point of possible phytotoxicity consists in interactions between nanomaterials and soil components and microorganisms, which significantly modify uptake of nutrients by plants [25]. The second point is closely connected with the uptake of nanomaterials (root, foliar), and their accumulation and transport within plant body with subsequent interactions with biomolecules, such as nucleic acids, proteins including enzymes, and cell structures, such as cell walls and biomembranes [21,26,27,28,29,30]. In particular the cell wall of plant cells represents a crucial structure in nanoparticle uptake compared to animal cells [31,32]. Application of nanoparticles with optical properties represent very efficacious tool in this area [33]. The third point is closely connected with their possible biotransformation, which has been demonstrated in desert plant species after application of ZnO nanoparticles, particularly, in soybean plants (*Glycine max*) after ZnO and CeO_2_ nanoparticles application or in *Prosopis* sp. plants after use of nickel hydroxide coated/uncoated nanoparticles [34,35,36]. Based on the results obtained, it can be concluded that foliar uptake of metal nanoparticles is connected with changes in leaf morphology and anatomy, especially of trichomes, stomata and hypodermis [37,38]. Root hairs represent the main site of uptake of nanoparticles from soils and aqueous media [28,39]. This fact is probably connected with endocytosis [40]. Nanoparticles are further transported into aerial parts of plants, including fruits [41,42]. Some plant organs show evidence of their accumulation according to chemical composition and surface modification [41,43,44]. Both positive and negative effects on plants have been demonstrated. Lu *et al.* demonstrated both positive and negative effects of nano-SiO_2_ and nano-TiO_2_ on nitrate reductase in soybean [45]. Enhancement of biomass production in spinach (*Spinacia oleracea*) after application of TiO_2_ nanoparticles has been recorded in a study by Gao *et al.* [46]. These changes are connected with the effects on nitrogen metabolism and photosynthesis [46]. Alumina nanoparticles mediated an enhancement of biomass accumulation in *Lemna minor*, which was based on the increased efficiency in the light reactions of photosynthesis [47]. On the other hand, some studies have proved the phytotoxicity of nanoparticles. TiO_2_ nanoparticles have been demonstrated to be toxic for the green alga *Desmodesmus* [48]. Zinc and ZnO nanoparticles inhibit germination of maize (*Zea mays*) and ryegrass (*Lolium perenne*), root elongation and function of radish (*Raphanus sativus*), rape (*Brassica napus*), ryegrass (*Lolium perenne*), lettuce (*Lactuca sativa*) and cucumber (*Cucurbita pepo*) [49]. Similar results have been obtained using Al_2_O_3_ nanoparticles and rare earth oxide nanoparticles [47,50]. Phytotoxicity has also been demonstrated in the case of plant treatment with sewage sludge containing multiwalled carbon nanotubes [51]. It is clear that mechanisms of nanoparticles phytotoxicity are widely discussed [52]. Phytotoxicity may be connected with the surface of nanoparticles, which can catalyse redox reactions in contact with biomolecules. This effect has been demonstrated in the case of carbon nanotubes [38,53]. Carbon nanotubes are able also directly affect function of some cells. Obstruction of stomata has been observed in the same study [38]. Application of nanoparticles may lead to oxidative stress, and formation of reactive oxygen species that are closely associated with damage of biomolecules. In addition, release of toxic ions from the surface of nanoparticles must be carefully considered [54]. Despite the above mentioned facts, there is only minimal knowledge about effect of nanoparticles on gene expression. Up-regulation of stress-related genes, such as *LeAqp2*, has been reported [30].

It is clear that studies focused on the effect of nanoparticles on biochemical markers of plants are almost completely missing, so we focused on the effect of iron oxide-based magnetic nanoparticles (MNPs) prepared by the method of plasma electrochemical deposition on plant cell model represented by tobacco BY-2 cell suspension culture in this study, which should reveal some biochemical pathways influenced by the particles.

## 2. Experimental Section

### 2.1. Chemicals

Reduced (GSH) and oxidized (GSSG) glutathione were purchased from Sigma-Aldrich (St. Louis, MO, USA). Phytochelatins 2, 3, 4, and 5 (PC2, PC3, PC4, PC5) were synthesized in Clonestar Biotech (Brno, Czech Republic) with a purity above 90%. HPLC-grade methanol (>99.9%; v/v) was from Merck (Dortmund, Germany) were used. All other chemicals used were purchased from Sigma Aldrich unless noted otherwise. Stock standard solutions of the thiols (1 mg/mL) were prepared with ACS water (Sigma-Aldrich) and stored in dark at −20 °C. Working standard solutions were prepared daily by dilution of the stock solutions. All solutions were filtered through 0.45 μm nylon filter discs (Millipore, Billerica, MA, USA) prior to high performance liquid chromatographic analysis. Acetate buffer of pH 5 was prepared with 0.2 M acetic acid and 0.2 M sodium acetate and diluted with water and used as a supporting electrolyte. 

### 2.2. Preparation of Deionised Water and pH Measurement

The deionised water was prepared using Aqual 25 reverse osmosis equipment (Aqua Osmotic, Tisnov, Czech Republic). The deionised water was further purified by using apparatus MiliQ Direct QUV equipped with the UV lamp. The resistance was 18 MΩ. The pH was measured using a WTW inoLab pH meter (Weilheim, Germany). 

### 2.3. Tobacco BY-2 Cell Suspension Culture and Microscopic Observations

The suspension culture of *Nicotiana tabacum* BY 2 line was grown in liquid Murashige and Skoog medium supplemented with sucrose (30 g/L), KH_2_PO_4_ (0.2 g/L), thiamine (1 mg/L) and 2,4-dichlorophenoxyaetic acid (0.2 mg/L) according to Nagata [55]. The suspension culture (20 mL) was cultivated in 50 mL Erlenmeyer flasks at 27 °C with shaking at 135 rpm (Kuhner Shaker, type: LT W, Adolf Kuhner AG, Basel, Switzerland). Subcultivation was performed after 3 or 4 days by transferring of 2 mL of suspension culture into a fresh cultivation medium (total volume 20 mL). One day after subcultivation in the exponential phase of growth, cells were treated with three concentrations of magnetic nanoparticles (0, 1, 10 and 100 µg/mL) for five days. Modified double staining with fluorescein diacetate (FDA) and propidium iodide (PI) for the determination of the viability of the cells was used [56,57]. In our experiments BY-2 cells (~1 mg) were harvested and diluted by fresh cultivation medium to the volume of 50 µL. The stock solutions of PI and FDA were added to a final concentration of 20 µg/mL and 1 µg/mL respectively. After 5 min of incubation at room temperature, the percentage of dead (red-stained cells) and viable cells (green-stained cells) was evaluated using an Olympus AX 70 fluorescence microscope with an Olympus cube U MWU coupled with the digital camera. The percentage quantification of red (dead cells) and green areas (viable cells) was determined in acquired digital picture by method IA (Image—Pro Plus was used, ver. 1.3, Sony, Tokyo, Japan). Acridine orange (AO, Sigma Aldrich, St. Louis, USA) was used as a general cytological stain, which is suitable for determination of lysozomal proton pump activity (in animal cells), changes in pH and detection of DNA/RNA. Working solution (5 µg/mL) was prepared by the diluting of stock solution (1 mg/mL, aq.) by in phosphate buffered saline (PBS) buffer (pH = 7.2). Cells were stained and incubated for 30 min at room temperature and dark. After it, cells were carefully washed by PBS buffer (pH = 7.2) and observed using fluorescence microscope (Axioskop 40, Carl Zeiss, Göttingen, Germany) equipped by wideband excitation and set of filters (FITC-DAPI, Carl Zeiss, Göttingen, Germany). Photographs were taken using digital camera (Olympus Camedia 750, Olympus, Tokyo, Japan). For the staining of thiols, 5-(bromomethyl)- fluorescein (5-BMF, Sigma-Aldrich, St. Louis, USA) was used. This probe reacts more slowly with thiols of peptides, proteins and thiolated nucleic acids in comparison with other fluorescent probes, but forms stronger thioether bonds that are expected to remain stable under the conditions required for fluorescence microscopy. Stock solution of 5-BMF (4 mM, anhydrous dimethyl sulfoxide) was prepared prior to staining because of the stability issues of 5-BMF. Working solution was prepared immediately using stock solution by diluting to final concentration of 20 µM (PBS buffer, pH = 7.6). Cells were incubated for one hour at room temperature and dark. After it, cells were carefully washed by PBS buffer (pH = 7.6) and observed using fluorescence microscope (Axioskop 40, Carl Zeiss) equipped by wideband excitation and set of filters (FITC-DAPI, Carl Zeiss, Göttingen, Germany). Photographs were taken using a digital camera (Olympus Camedia 750, Olympus, Tokyo, Japan). Determination of growth parameters was based on determination of fresh weight (FW). Samples were collected in strictly defined time intervals, washed with fresh cultivation MS medium and weighed (Sartorius R160P, Sartorius GmbH, Goettingen, Germany). 

### 2.4. Magnetic Nanoparticles, Their Characterization and Modification

Magnetic nanoparticles (MNPs) used in our experiments were prepared by the plasma-enhanced chemical vapour deposition method (PECVD) [58]. Their chemical composition responded to maghemite (γ-Fe_2_O_3_). Their size was 24 nm. In addition, we prepared also MNPs modified by water vapour (MNPs-OH) and ammonia vapour (MNPs-NH_2_). Crystallic Fe_2_O_3_ phase was determined using X-Ray structural analysis. Their size characterisation was performed using scanning electron microscope (Tescan, Brno, Czech Republic).

### 2.5. Sample Preparation

#### 2.5.1. Spectrophotometric Measurements

Harvested and washed BY-2 cells (approximately 0.1 g of fresh weight) were transferred to a test-tube of 2 mL volume (Eppendorf, Hamburg, Germany). The samples were frozen by liquid nitrogen to disrupt the cells. The frozen sample was further homogenised using an Ultra-Turrax T8 homogenizer (IKA, Staufen, Germany). Then, 1 mL of 0.2 M phosphate buffer (pH = 7.0) was added and the sample was homogenized for 5 min. After it, homogenate was quantitatively transferred to a new test-tube. The mixture was further homogenised by shaking on a Vortex–2 Genie (Scientific Industries, New York, NY, USA) at 4 °C for 15 min. The homogenate was centrifuged (16,000 g) for 15 min at 4 °C using a Universal 32 R centrifuge (Hettich-Zentrifugen GmbH, Tuttlingen, Germany). Supernatant was filtered through a membrane filter (0.45 μm nylon filter disk, Millipore) prior to analysis.

#### 2.5.2. Chromatographic Measurements

An amount of approximately 0.5 g of washed BY-2 cells was frozen using liquid nitrogen and subsequently homogenized with 0.5 mL of potassium buffer (pH 7.0) using the Ultra-Turrax T8 at 25,000 rpm for 3 minutes. In addition, 0.5 mL of potassium buffer was added. The homogenate was centrifuged (15,000 g) for 15 min at 4 °C using the Universal 32 R centrifuge. Supernatant was collected and used for chromatographic analysis.

### 2.6. Spectrophotometric Measurements

#### 2.6.1. Determination of Total Protein Content—Pyrogallol Method

Reagent R1 (100 mM succinic acid, 6.94 mM sodium benzoate, 0.12 mM sodium molybdate, 2.09 mM sodium oxalate) in a volume of 200 µL was pipetted into plastic cuvette for protein determination. Furthermore, 20 µL of sample was added. Pyrogallol red with sodium molybdate is bound in the complex with proteins in a succinic buffer at pH 2.5. This complex results in a shift of the absorption peak from 460 nm (agent) to 600 nm (complex). The absorption at wavelength λ = 605 nm was measured after 10-min incubation at 37 °C. The absorbance values were used to calculate the absorption of reagent itself and absorbance values after 10 minutes of incubation with the sample.

#### 2.6.2. Determination of Total Thiol Compounds and Glutathione-*S*-Transferase Activity

##### 2.6.2.1. Total Thiols—Ellman’s Reaction

Ellman’s spectrophotometric method was used for the determination of sulfhydryl (-SH) groups [59]. Ellman’s reagent (277 µL, reagent 1, R1—2 mM 5,5’-dithiobis(2-nitrobenzoic) acid (DTNB) in 50 mM Na_2_(CH_3_COO)_2_) was mixed with a sample (45 µL). After it, 33 µL of reagent R2 (1 M trisma base-CH_3_COOH) was added. Mixture was incubated for 10 min at 37 °C, absorbance was measured at λ = 405 nm. Values of absorbance of reagent R1 itself (blank) and mixture after 10-min incubation were used for the calculation of total -SH content. 

##### 2.6.2.2. Glutathione-S-Transferase Activity

The method is based on the glutathione-*S*-transferase (GST)-catalysed reaction between GSH and GST substrate, 1-chloro-2,4-dinitrobenzene (CDNB), which has the broadest range of isozyme detectability (e.g., alpha-, mu-, pi- and other GST isoforms). Under certain conditions, the interaction between glutathione and CDNB is very dependent on the presence of active GST. The GST-catalysed formation of GS-DNB produces a dinitrophenylthioether, which can be detected spectrophotometrically at 340 nm. The volume of 180 µL reactants consisted of 2 mM CDNB in PBS (1:19, *v*/*v*, 37 °C) was added to a sample of volume of 40 µL. After it, 30 µL of 12.5 mM GSH in 0.1 M phosphate buffer (pH = 7.4) was added. A microtube was carefully stirred and loaded into an automatic biochemical analyser BS-200 (Mindray, Shenzhen, China) and measured at 340 nm.

#### 2.6.3. Determination of Antioxidant Activity

An automated BS-400 spectrophotometer (Mindray) was used for determination of antioxidant activity according to the following protocols. 

##### 2.6.3.1. DPPH Test

A volume of DPPH^•^ reagent (200 μL) was incubated with sample (20 μL). Absorbance was measured after 15 min of incubation at λ = 510 nm. For calculating the antioxidant activity value of absorbance of reagent itself (A_0_) and the value of absorbance after 15 min of incubation (A_15_) were used. Resulting value was calculated according to the formula: A = A_15_ − A_0_ [60]. 

##### 2.6.3.2. ABTS Test

A volume of ABTS^•^ reagent (245 μL) was pipetted into a plastic cuvette with subsequent addition of sample (5 μL). Absorbance was measured at λ = 670 nm after 15 min. For calculating the antioxidant activity, we used the value of absorbance of reagent itself (A_0_) and the value of absorbance after 15 min of incubation (A_15_). Resulting value was calculated according to the following formula: A = A_15_ − A_0_ [60]. 

##### 2.6.3.3. DMPD Method

A volume of DMPD^•^ reagent (200 μL) was pipetted into a plastic cuvette. Then, sample (5 μL) was added. Absorbance was measured at λ = 510 nm for 15 min. For calculating the antioxidant activity, the value of absorbance of reagent itself (A_0_) and the value of absorbance after 15 min of incubation (A_15_) were used. Resulting value was calculated according to the following formula: A = A_15_ − A_0_ [60]. 

##### 2.6.3.4. Blue CrO_5_ Method

A volume of CrO_5_ reagent (400 μL) was pipetted into a plastic cuvette. After it, sample (4 µL) was added. Absorbance was measured at 546 nm after 192-sec incubation (A_1_). Then, the second reagent was added (40 µl). Absorbance was measured at the same wavelength after 192-sec incubation (A_2_). Resulting value of antioxidant activity was calculated according to the following formula: A = A_2_ – A_1_ [60].

### 2.7. High Performance Liquid Chromatography with Electrochemical Detection (HPLC-ED)—Determination of Thiol Compounds

The system consisted of a solvent delivery pump operating in range of 0.001–9.999 mL/min (Model 582 ESA Inc., Chelmsford, MA, USA), a guard cell (Model 5020, ESA), a chromatographic column (Polaris C18-A, 4.6 mm, 5 µm particle size), and an electrochemical detector. The electrochemical detector (ED) includes one low volume flow-through analytical cell (Model 5040, ESA), which is consisted of glassy carbon working electrode, palladium electrode as reference electrode and auxiliary carbon electrode, and Coulochem III as a control module. The sample (5 μL) was injected manually. The obtained data were processed by CSW 32 software. The experiments were carried out at room temperature. Guard cell potential was 0 V. A glassy carbon electrode was polished mechanically by 0.1 μm of alumina (ESA) and sonicated at room temperature for 5 min using a Sonorex Digital 10 P Sonicator (Bandelin, Berlin, Germany) at 40 W. 

### 2.8. Mathematical Treatment of Data and Estimation of Detection Limits

Data were processed using Microsoft Excel^®^ (Richmond, WA, USA) and STATISTICA.CZ Version 8.0 (StatSoft, Prague, Czech Republic). Results are expressed as mean ± standard deviation (S.D.) unless noted otherwise (Excel^®^). Statistical significances of the differences were determined using STATISTICA.CZ. Differences with *p* < 0.05 were considered significant and were determined by using of one way ANOVA test (particularly the Scheffe test), which was applied for means comparison. 

## 3. Results and Discussion

Presently, nanoparticles (NPs) have found use in different branches of industry and medicine. However, data about their toxicity and potential risk for the environment are still scarce. Due to this fact, we focused on studying of the effect of magnetic γ-Fe_2_O_3_ nanoparticles (NPs) and modified magnetic nanoparticles (MNPs—Fe_2_O_3_-OH and Fe_2_O_3_-NH_2_) on tobacco BY-2 plant cell suspension culture in this study. Cells were treated with the above mentioned nanoparticles in concentrations of 0 (control), 1, 10 and 100 ng/mL for 120 h. After it, they were collected and submitted to analyses. We aimed at determining the effect of NPs and MNPs on growth of cell suspension culture and its viability, proteosynthesis—total protein content, protective thiol compounds—total thiols, content of reduced glutathione (GSH), oxidized glutathione GSSG, phytochelatins 2-5, and glutathione S-transferase (GST) activity. In addition, we also monitored the effect of NPs and MNPs on antioxidant activity of BY-2 cell extracts. Changes in cell architecture and viability and total thiol content were determined using fluorescent staining.

### 3.1. Effect of NPs and MNPs on Cell Viability and Growth

Application of both NPs and MNPs led to the changes in cell viability and cell growth. However, there were significant differences between NPs and MNPs. Whereas non-modified MPs had no significant effect on BY-2 cell viability (96% after 120-h treatment under the highest concentration of 100 ng/mL compared to control—99%), application of both -OH and -NH_2_ modified NPs led to a significant reduction of cell viability at all concentrations. At the lowest concentration (1 ng/mL), viability of BY-2 cells was reduced to 62.5% (Fe_2_O_3_-NH_2_) and 75% (Fe_2_O_3_-OH) after 120-h treatment. Treatment of BY-2 cells with MNPs in the highest concentration (100 ng/mL) resulted in a reduction of viability to 45 % for Fe_2_O_3_-NH_2 _and to 60% for Fe_2_O_3_-OH. These results confirm the toxicity of MNPs and indicate the necessity to discuss possible modifications of NPs in living organisms, when they can contribute to enhancement of NP toxicity. Questions about bioavailability of nanoparticles remain almost unanswered and must be further investigated, especially with a view to their potential risk to living organisms. Uptake of nanoparticles has been demonstrated for carbon nanotubes, quantum dots and some other nanoparticles types; however, available data are still very limited [54,61,62,63]. Stability of NPs and possible release of free metal ions must be also carefully considered, especially due to potential toxicity of free metal ions. On the other hand, there are many enzymes using heavy metal ions as cofactors of ubiquitous enzymes [64]. The questions focused on possible catalytic activity of NPS surface due to interactions with enzymes stay unknown and must be further investigated. 

**Figure 1:**
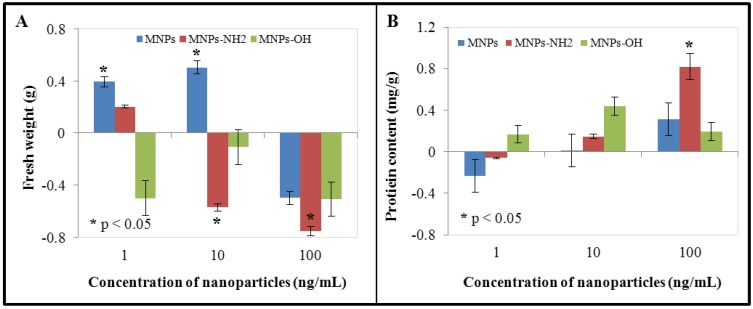
The influence of 0, 1, 10 and 100 ng/mL of magnetic γ-Fe_2_O_3_ nanoparticles (NPs) and modified magnetic nanoparticles (MNPs—Fe_2_O_3_-OH and Fe_2_O_3_-NH_2_) on (**A**) fresh weight and (**B**) protein content of treated tobacco BY-2 cells. The results were subtracted from the control, *i.e.*, non-treated BY-2 tobacco cells. The cells were treated for five days.

The second step consisted in determination of the effects of NPs and MNPs on growth of BY-2 cell suspension culture. Application of NPS and Fe_2_O_3_-NH_2 _caused lower enhancement of fresh weight (FW) in the lowest concentration (1 ng/mL) in comparison with control untreated BY-2 cells (for 7% to 107% in the case of NPS and almost for 4% to 104% for Fe_2_O_3_-NH_2_). All other experimental variants demonstrated growth depression compared to control. The most evident growth depression was observed in the case of Fe_2_O_3_-NH_2 _in the highest concentration (100 ng/mL), where FW was reduced to 85% in comparison with untreated BY-2 cells. Values for other experimental variants in the highest concentration were as follows: 91% (MPs) and 90% (Fe_2_O_3_-OH) (Figure 1(A)). 

These growth changes were not so accentuated in comparison with treatment of BY-2 cell suspension culture by heavy metal ions or by organic compounds including plant secondary metabolites [65,66,67,68,69]. On the other hand, concentration of used nanoparticles was very low (to the limit of 100 ng/mL, respectively 100 µg/L). In addition, determination of growth parameters by weighing may be misrepresented by the presence of fragments of death and necrotized cells. In our case, collected cells were carefully washed by fresh cultivation medium, which means that all small cell fragments were removed. In the case of recalculation of obtained results of FW to cell viability and determined water content, we can say that application of NPs and MNPs led to enhancement of the water content in cells, which was 63% for control, 65% for NPS (103.2 % of control), 72% for Fe_2_O_3_-OH (114.3% of control) and 74% for Fe_2_O_3_-NH_2 _(119.4% compared to control) in the highest applied concentration (100 ng/mL, not shown). This fact may be connected with possible interactions of the surface of MNPs with molecules of water. However, further details are still missing and must be further investigated.

### 3.2. Effect of NPs and MNPs on Protein Content

Treatment of BY-2 cells by both NPs and MNPs led to the enhancement of protein content at the two highest concentrations (10 and 100 ng/mL) in comparison with control. On the other hand, at the lowest concentration, protein content was slightly reduced in the case of NPs and Fe_2_O_3_-NH_2_. Fe_2_O_3_-OH MNPs demonstrated enhanced protein content in all experimental variants. Compared to control, values of protein content in cells treated with Fe_2_O_3_-NH_2 _and Fe_2_O_3_-OH were as follows: 78%, 94% and 116% (1 ng/mL), 102%, 114% and 142% (10 ng/mL) and 129%, 178% and 118% (100 ng/mL, Figure 1(B)). Proteins play many crucial roles in cells. They have structural, transport or catalytic (enzymes) function. In addition, there are peptides/proteins with special functions, such as glutathione and phytochelatins and enzymes involved in biochemical pathways connected with these compounds, which play crucial role in detoxification of both heavy metals and organic pollutants—xenobiotics [70,71,72,73]. Enhanced amount of total proteins may be based on the ability of NPs and MNPs to induce protective cell mechanisms, such as biosynthesis of protective peptides/proteins and enzymes, which are involved in detoxification processes. In addition, interactions of NPs and MNPs surface with macromolecules may be expected; however, these questions remain unanswered. 

### 3.3. Effect of NPs and MNPs on Intracellular Thiols

**Figure 2:**
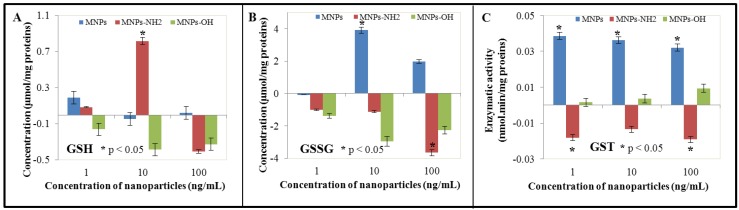
The influence of 0, 1, 10 and 100 ng/mL of magnetic γ-Fe_2_O_3_ nanoparticles (NPs) and modified magnetic nanoparticles (MNPs—Fe_2_O_3_-OH and Fe_2_O_3_-NH_2_) on (**A**) concentration of reduced glutathione (GSH), (**B**) concentration of oxidized glutathione (GSSG) and (**C**) glutathione *S*-transferase activity (GST) of treated tobacco BY-2 cells. The results were subtracted from the control, *i.e.*, non-treated BY-2 tobacco cells. The cells were treated for five days.

Thiol compounds as reduced and oxidized glutathione represent the first protective substances against heavy metal ions and other pollutants. They are able to maintain redox status of cells. In addition, these compounds are involved in transport of heavy metal ions/xenobiotics and their further compartmentation. Due to the above mentioned facts, we focused on the ability of NPs and MNPs to induce biosynthesis of reduced (GSH) and oxidized (GSSG) glutathione. In addition, we monitored activity of glutathione *S*-transferase (GST), group of enzymes with different cell localisation (cytosolic, mitochondrial and microsomal). It is well known that these proteins/enzymes can constitute up to 10% of all cytosolic proteins [74]. Function of GST consists in catalysis of conjugation reactions of reduced glutathione (GSH) with a wide range of electrophilic substrates [73]. Firstly, we determined total thiol content, which was in highest concentrations of NPs and Fe_2_O_3_-NH_2_ reduced and reversely enhanced in Fe_2_O_3_-OH in comparison with control BY-2 cells. The most significant reduction was observed in the case of Fe_2_O_3_-NH_2_, where thiol content reached only 56% of value determined in control untreated BY-2 cells. However, in the case of other concentrations, differences in total thiol content were only moderate. The most significant enhancement of total thiol content was observable in the case of NPs in the lowest concentration—1 ng/mL. This enhancement was for almost 32% (comparing values determined for Fe_2_O_3_-NH_2_—enhancement for 9% and for Fe_2_O_3_-OH—reduction for 4%). However, the thiol compounds group includes not only glutathione and phytochelatins, but also other compounds, such as the amino acid cysteine, which is crucial for biosynthesis of glutathione, but also all proteins containing -SH groups, which are crucial in protein folding and formation of the tertiary protein structure via disulphide bonds. In addition, there are a lot of cofactors, whose structure contains thiol group/groups. The second step consisted in determination of reduced (GSH) and oxidized (GSSG) glutathione and their reciprocal rate, which inform us about GSH utilization in detoxification processes. Obtained values were recalculated to total protein content and related to control (Figure 2(A,B)). Whereas the lowest NPs and MNPs concentration led to only moderate changes in GSH content (NPS—enhancement for 24.5%, Fe_2_O_3_-NH_2_—enhancement for 11.2%, and Fe_2_O_3_-OH—reduction for 20.9% to 79.1%), changes caused by the two highest concentrations were more evident. Application of Fe_2_O_3_-NH_2 _led to the enhancement for almost 106% to 206% at the concentration of 10 ng/ml and, on the contrary, to reduction for almost 54% to 47.3% of value determined in control, untreated variant in the highest concentration—100 ng/mL. Reduction of GSH amount was observed at all concentrations of Fe_2_O_3_-OH. This reduction was the most significant in the concentration 10 ng/mL. This interesting phenomenon can be associated with that fact that GSH plays a key role in detoxification of xenobiotics with reactive moieties such as -OH moieties. The interaction of Fe_2_O_3_-OH with GSH could lead to depletion of the reduced glutathione, which is clearly evident on the decreasing content of GSH with the increasing content of the particles. In the case of non-modified nanoparticles, detected values of GSH were comparable with control variant, significant enhancement was determined in the lowest concentration (Figure 2(A)). On the other hand, changes in oxidized glutathione were clearly evident for all experimental concentrations with exception of NPs at the lowest concentration (1 ng/mL), where GSSG content was almost the same, which was detected in control BY-2 cells (Figure 2(B)). Distinctive GSSG reduction was observed in the case of both Fe_2_O_3_-NH_2_ and Fe_2_O_3_-OH nanoparticles, whereas this reduction was concentration-dependent. Application of Fe_2_O_3_-NH_2 _led to the GSSG reduction for 18.2% to 81.8% in comparison with control (1 ng/mL), for 40% to 60% of control (10 ng/L) and for 65% to 35% (100 ng/mL) of the value determined in control experiments. In the case of Fe_2_O_3_-OH MNPs, these values were as follows: 75.1% (1 ng/mL, reduction for 24.9% compared to control), 47.6% (10 ng/mL, reduction for 52.4% compared to control), and 59.6% (100 ng/mL, reduction for 40.4% compared to control). Quite different results were obtained in the case of non-modified NPs, where GSSG enhancement was well evident. Its values reached 169.4% of control (10 ng/mL) and 135.1% of control (100 ng/mL). At the lowest concentration, slight GSSG reduction was observable (to 98.4% of control). Activity of glutathione *S*-transferase was significantly enhanced in the case of non-modified NPs and Fe_2_O_3_-OH nanoparticles; however, whereas GST activity diminished with increased NPs concentration (from 211% (1 ng/mL) to 191% (100 ng/mL) of control), GST activity increased with increased Fe_2_O_3_-OH concentration (from 105% (1 ng/mL) to 129% (100 ng/mL) of control]. Application of Fe_2_O_3_-NH_2_ nanoparticles led to the accentuated reduction of GST activity in comparison with control (48.6%, 62.8%, and 45.7% of control, Figure 2(C)). 

Whereas the role of iron in plant biochemistry is well known, the effect of nanoparticles based on iron oxides on plant cells is still unknown. Iron ions induce biosynthesis of glutathione, therefore, glutathione seems to be important in iron metabolism in cells [75]. However, these data have been confirmed on animal cell models or on animals under experimental conditions. Connection of glutathione and iron is discussed in pathogenesis of some diseases including cancer [76,77,78]. The role of glutathione in iron metabolism in plants is still subject to discussion. Iron deficiency enhances levels of ascorbate, glutathione, and other related enzymes in sugar beet roots (*Beta vulgaris*, *Amaranthaceae*) and levels of GST-like enzyme in alfalfa (*Medicago sativa, Fabaceae*) [79,80]. Iron excess leads to cytotoxic effects. This effect is enhanced by the presence of other metal ions, such as aluminium [81]. It seems that glutathione plays an important protective role in cytotoxicity induced by iron ions. This fact may explain the obtained results—increased levels of thiols and GSH at the lowest concentration of applied nanoparticles and their reduced levels at the two highest NPs and MNPs concentrations. Reduction of GSH levels in this case may be caused by release of free iron ions from the nanoparticles’ surface, which are subsequently bound in complexes with glutathione. 

GSH/GSSG rates inform us about possible oxidative stress in cells. Accumulation of GSSG is closely connected with oxidative stress [82]. Glutathione is one of the most important antioxidants, which detoxify ROS and protect plants against oxidative stress [72,83]. When acting as an antioxidant, reduced glutathione (GSH) is oxidized to oxidized glutathione (GSSG), so, antioxidant activity is attributed to the reduced glutathione form. Therefore, it is necessary for plants to maintain high GSH/GSSG rates. There are mechanisms involved in GSH maintenance. One of the most important mechanisms consists in action of enzyme glutathione reductase (GR), which catalyses reduction of GSSG to GSH with the NADPH as a donor of electron. This process takes place in glutathione-ascorbate cycle. It has been demonstrated that GSSG accumulation is closely connected with metabolism of other compounds, however, this fact has been confirmed in animal cell models. GSH/GSSG rate can significantly affect the redox homeostasis in cells and subsequently formation of disulphide bonds in proteins. Reciprocal GSH/GSSG rate is important for plant acclimatization, however, it plays crucial role in resistance to abiotic stress, such as increased salinity, low temperatures or excess of heavy metals, such as zinc or cadmium [84,85]. The GSH/GSSG rate determined was 0.137 in control BY-2 cells. At the lowest concentration (1 ng/mL), the lowest GSH/GSSG rate was detected in the case of Fe_2_O_3_-OH nanoparticles (0.144); however, this rate was still higher in comparison with control. At the middle concentration (10 ng/mL), the most reduced GSH/GSSG rate was detected in non-modified nanoparticles to be only 0.077. The lowest GSH/GSSG rate was detected also in the case of non-modified nanoparticles at the highest concentration (100 ng/mL). These results indicate the ability of non-modified nanoparticles to induce oxidative stress. The question why modification of the NPs surface leads to the reduction of their ability to induce oxidative stress remains. The most probable mechanism of induction of oxidative stress in based on the reaction between iron(II) ion and hydrogen peroxide molecules resulting in formation of highly toxic and reactive hydroxyl radical and iron(III) ion. This radical is responsible for damage to biomolecules under loss of their function. Iron(III) ion may be reduced to iron(II) ion by hydrogen peroxide with formation of peroxide radicals and protons. Magnetic γ-Fe_2_O_3_ nanoparticles are represented by iron(III) oxide in a specific arrangement. The possibility of participation of iron(III) in regeneration of iron(II) under formation of peroxide radical in the presence of hydrogen peroxide remains still unknown and must be further carefully considered. Peroxide radical as well as other types of radicals may be scavenged by polyphenols in plants. In conclusion, GSH as well as polyphenols in plants are responsible for antioxidant properties and defence against oxidative stress. Due to this fact, we were focused on determination of antioxidant activity of untreated as well as NPS- and MNPs-treated variants. This part of experimental work will be introduced and discussed in an independent sub-section. 

**Figure 3:**
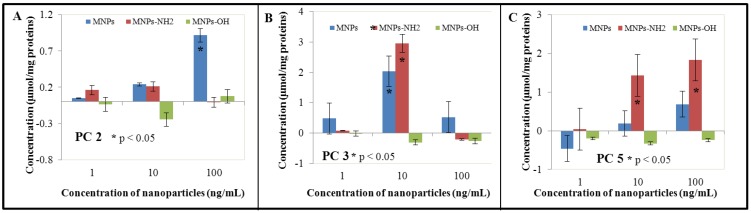
The influence of 0, 1, 10 and 100 ng/mL of magnetic γ-Fe_2_O_3_ nanoparticles (NPs) and modified magnetic nanoparticles (MNPs—Fe_2_O_3_-OH and Fe_2_O_3_-NH_2_) on (**A**) concentration PC2, (**B**) concentration PC3 and (**C**) concentration PC5 in treated tobacco BY-2 cells. The results were subtracted from the control, *i.e.*, non-treated BY-2 tobacco cells. The cells were treated for five days.

We were focused not only on GSH, GSSH and GSH/GSSG rate in our work, but also on phytochelatins (PCs), oligomers of glutathione with general structure of (γ-glutamyl-cysteinyl)_n_-glycine (n = 2–11), which are synthesized by phytochelatin synthase. They were firstly discovered as cadmium-binding cadystins A and B in a fission yeast and then in many plants [70,86,87]. Whereas the role of PCs in intact plants is discussed in connection with transport and compartmentation of heavy metal ions, such as cadmium, role of PCs in cell suspension cultures remains almost unknown. Works that demonstrate formation of cadmium-PCs complexes have been published, so, there is a strong evidence of PCs’ role in cadmium tolerance in plant cell suspension cultures [88,89,90]. Similar results have also been published for other heavy metals, such as inorganic arsenic, copper, and zinc. We focused on the biosynthesis of three PCs, PC2, PC3, PC4 and PC5, in connection with NP and MNP treatment in our study (Figure 3). Generally, differences between concentration of nanoparticles, type of modification and type of PC were observed. Generally, NPs and Fe_2_O_3_-NH_2 _induced PCs biosynthesis. These trends were observable for almost all monitored PCs—PC2, PC3 and PC5. Different results were obtained in the case of PC4 for Fe_2_O_3_-NH_2 _nanoparticles, where their application led to distinctive reductions of PC4 amount at all applied concentrations (62.2% (1 ng/mL), 67.3% (10 ng/mL) and 51.2% (100 ng/mL) of value detected in untreated BY-2 cells). On the other hand, treatment of BY-2 cells by Fe_2_O_3_-OH led to the reduction of PCs in total. This fact was well evident in the case of PC2, PC3 and PC5, whereas level of PC4 was enhanced in applied concentrations of 1 ng/mL (to 136.2% of control) and 100 ng/mL (to 170.4% of control). Contrariwise, a middle concentration (10 ng/mL) of Fe_2_O_3_-OH caused reduction of PC4 level for 12.9% to 87.1% of value determined in untreated control variant.

It is well evident that the obtained data must be carefully considered in connection with the treatment of experimental BY-2 cells by NPs and MNPs. Firstly, enhancement of PCs may be connected with possible toxic effect of iron ions. However, there is almost no evidence about induction of PCs biosynthesis induced by iron ions in the literature. The work of Bhuyian *et al.* demonstrates enhancement of heavy metal tolerance in transgenic *Brassica juncea* (*Brassicaceae*) with introduced *AtATM3* gene [91]. AtATM3 is localised at the mitochondrial membrane of *Arabidopsis thaliana* (*Brassicaceae*) and is closely associated with biogenesis of Fe-S clusters and iron homeostasis. However, introduction of *AtATM3* gene led to the enhanced tolerance to heavy metal ions by induction of expression of several metal transporters. In addition, AtATM3 transgenic *Brassica juncea* demonstrated also higher expression of *BjPCS1* (phytochelatin synthase 1). This work shows evidence of a connection of PC biosynthesis induction in connection with iron ions. Similar results were obtained in *Lotus japonicus* (*Fabaceae*)*,* where the ability of iron to induce phytochelatin synthase 3 (LjPCS3) was detected [92]. Induction of phytochelatin synthase (SmPCS) by iron ions was detected in *in vitro* culture of *Schisostoma mansonii*, however, this species belongs to the kingdom *Animalia* [93]. Different results are presented in the work of Loscos *et al.*, where the role of phytochelatin synthase contribution in detoxification of iron is not confirmed [94]. Secondly, the large surface areas of nanoparticles must be discussed not only with the view to their potential toxicity, but also with possible interactions with other metal ions. It is well known that cultivation media contain both macroelements and microelements, especially metal ions. They play essential roles in plant nutrition, respectively in many biochemical and physiological processes. Microelements are widely discussed as cofactors of many enzymes. Their excess leads to the symptoms of toxicity. Can NPs modify the uptake of other metal ions? Answers are still missing. Only primary data have been published in the case of TiO_2_ nanoparticles, where application of lead acetate increased toxicity of TiO_2_ nanoparticles in mice [95]. However, data about synergic/antagonistic effect of nanoparticles and metal ions on plants are missing and the potential role of NPs and MNPs in uptake of other metal ions must be further investigated. 

### 3.4. Effect of NPs and MNPS on Antioxidant Activity

**Figure 4:**
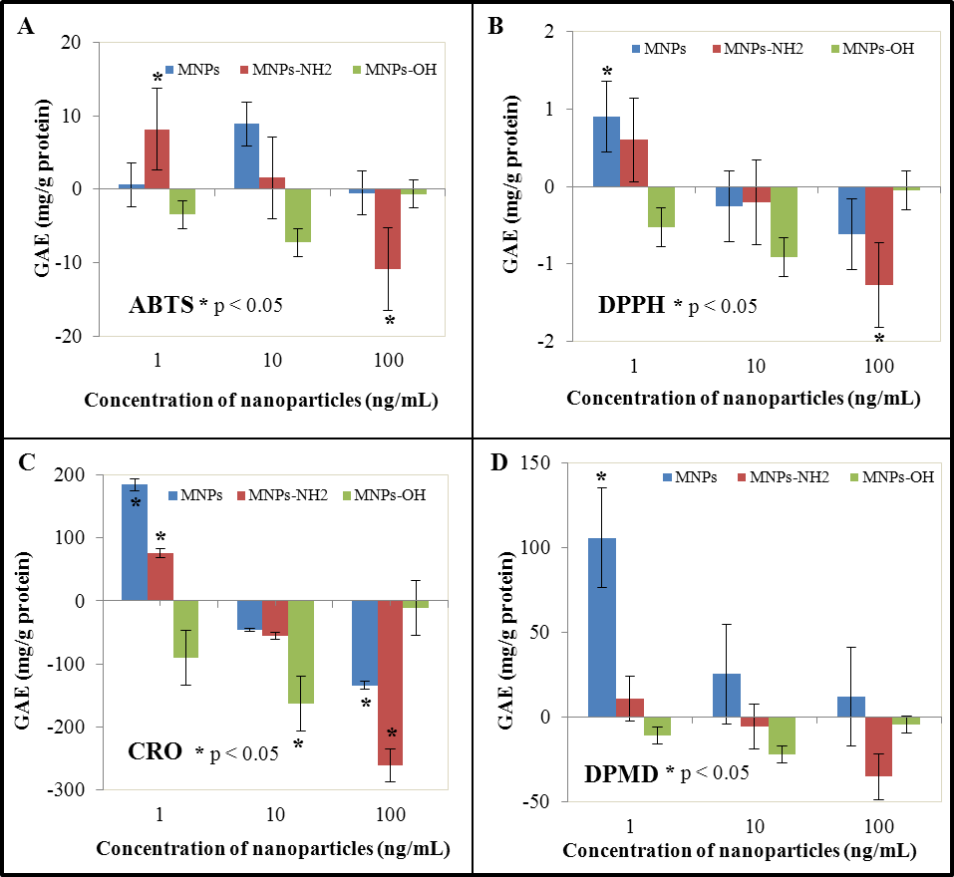
The influence of 0, 1, 10 and 100 ng/mL of magnetic γ-Fe_2_O_3_ nanoparticles (NPs) and modified magnetic nanoparticles (MNPs–Fe_2_O_3_-OH and Fe_2_O_3_-NH_2_) on antioxidant activity of treated tobacco BY-2 cells measured by (**A**) ABTS, (**B**) DPPH, (**C**) CRO and (**D**) DPMD assay. The results were subtracted from the control, *i.e.*, non-treated BY-2 tobacco cells. The cells were treated for five days.

Compounds with antioxidant activity play an important role in protection against reactive oxygen species (ROS), as well as reactive nitrogen species (RNS). The ability of different compounds to induce generation of ROS/RNS is well known. Especially heavy metal ions are among the most potent ROS inducers. Various methods have been developed and used in the determination of the antioxidant activity of different compounds or extracts. Analytical methods are based on the determination of the radical-scavenging activity of antioxidants against free radicals, such as the 1,1-diphenyl-2-picrylhydrazyl (DPPH) radical. The DPPH method has been developed for determination of superoxide anion radical, hydroxyl radical and peroxyl radical scavenging activity [96]. The ABTS method is based on the 2,2′-azinobis(3-ethylbenzothiazoline)-6-sulfonic acid (ABTS) radical cation, which is used to screen the flavonoids and polyphenolics radical scavenging activity [97]. The DMPD method uses a buffered solution of N,N-dimethyl-*p*-phenylenediamine (DMPD) in acetate buffer. The DMPD radical cation is subsequently reduced by hydrogen-donating antioxidants [98]. This method was used for determination of antioxidant activity of both phenolics and biological matrices, such as fruits, cereal or wine [99,100]. The blue CrO_5_—assay (CRO) was developed for determination of the antioxidant activity of a wide range of substrates. Chromium peroxide is produced from ammonium chromate in an acidic environment in the presence of hydrogen peroxide [101]. This method shows high sensitivity, linearity, and repeatability. The above mentioned methods were used in our experiments to determine the antioxidant activity of extracts of BY-2 cells under NPs and MNPs treatment. Their application led to the significant reduction of antioxidant activity at the two highest concentrations (10 and 100 ng/mL) in the case of all used nanoparticles; on the other hand, antioxidant activity was enhanced almost in all cases at the lowest concentration (1 ng/mL), with the exception of Fe_2_O_3_-OH nanoparticles. Fe_2_O_3_-OH nanoparticles showed a reduction of antioxidant activity in all experimental variants (Figure 4). This decrease can be considered as a proof of our presumption that Fe_2_O_3_-OH bears the most reactive moiety, which interacts with other biological molecules. This is in very good agreement with the results obtained by determination of GSH (Figure 2(A)). 

The most accentuated enhancement of antioxidant activity was detected at the lowest concentration (1 ng/mL) of NPs using the DMPD and CRO methods (to 238.5%, respectively to 132.8% of control). On the other hand, the most significant reduction of antioxidant activity was proved in the case of Fe_2_O_3_-NH_2_ at the highest concentration using the CRO method (reduction to 53.9% of control). These facts indicate the ability of NPs as well as MNPs to generate reactive oxygen species with subsequent initiation of protective mechanisms including biosynthesis of protective antioxidant compounds. These compounds include both intracellular thiols (GSH) and other compounds, such as polyphenolics. The abovementioned results are in agreement with detected levels of GSH—in the lowest applied NPs and MNPs concentration, induction of GSH production—enhancement of GSH levels—was observed with exception of the Fe_2_O_3_-OH nanoparticles, where GSH level was reduced also in the lowest applied concentration. These data are in agreement with data obtained by measurement of antioxidant activity of BY-2 cell extracts. Contrariwise, in comparison of PCs levels and levels of antioxidant activity, it is obvious that PCs do not contribute to antioxidant activity.

### 3.5. Microscopical Observations

**Figure 5:**
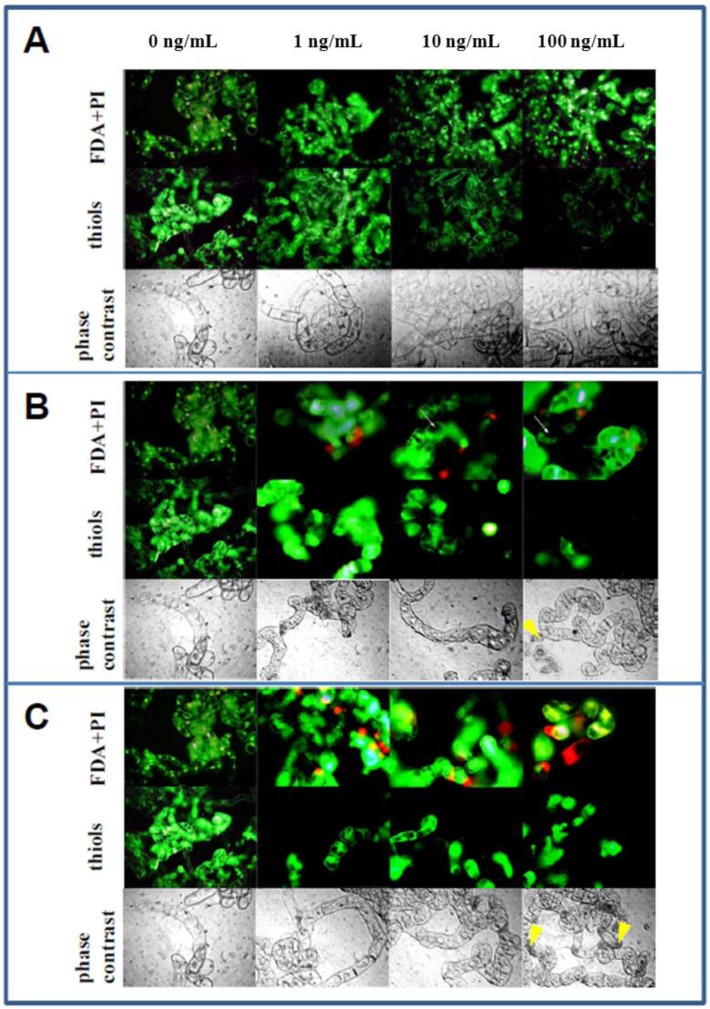
Microscopic observations of tobacco BY-2 cells treated with 0, 1, 10 and 100 ng/mL of (**A**) magnetic γ-Fe_2_O_3_ nanoparticles (NPs) and modified magnetic nanoparticles ((**B**) Fe_2_O_3_-NH_2_ and (**C**) Fe_2_O_3_-OH). The cells were treated for five days.

BY-2 cells treated with NPs and MNPs were subjected to microscopical observation (Figure 5). Despite the fact that FDA/PI staining served for determination of BY-2 cell viability based on the conversion of FDA to fluorescein, combination of these two fluorescent probes appeared to also be suitable for cytological observations [56,102]. Application of non-modified NPs didn’t lead to observable changes in cell architecture, which was comparable with the cell architecture of untreated BY-2 cells (Figure 5(A)). The cytoplasm did not manifest characters of shrinkage, which is typical for cells undergoing programmed death. Control and NPs-treated BY-2 cells had clearly bounded vacuoles without signs of permeability disruption (Figure 5). Different cell structure was observed in both Fe_2_O_3_-NH_2_ and Fe_2_O_3_-OH treatment (Figure 5 (B,C), respectively), especially at the two highest concentrations (10 and 100 ng/mL). Cells were more vacuolised, and in many cases cytoplasm formed only a thin layer along the cell wall/plasmalemma. In addition, a relatively high rate of BY-2 cells was diffusely engrained by fluorescein, which indicates disruption of plasmalemma/tonoplast semi-permeability. These changes were evident mostly in the Fe_2_O_3_-OH treated variants. 

Staining by acridine orange (AO), which is useful for general cytology as well as detection of programmed cell death/apoptosis and pH changes, enabled detection of cells with lowered cytosolic pH values, especially in variants treated with Fe_2_O_3_-OH. These changes may indicate subsequent processes of programmed cell death in plant cells [103]. Whereas cytoplasm shrinkage was observed in Fe_2_O_3_-OH and Fe_2_O_3_-NH_2_ variants, fragmentation of cell nuclei and formation of apoptotic-like bodies were not evident. These facts were confirmed using the method of phase contrast. We were focused also on localization of thiol compounds. For this purpose, 5-(bromomethyl)fluorescein was used (measurement of thiol-containing amino acids and phytochelatin (PC2) via capillary electrophoresis with laser-induced fluorescence detection). There were significant differences between BY-2 cells at different ontogenetical stages. Whereas young cells demonstrated high emission, older and old cells with big central vacuoles showed reduced emission. These changes are probably connected with the depletion of the thiol pool in these cells due to NP and MNP treatment. On the other hand, ontogenetically young cells may represent cells, which were selected on media supplemented by NPs and MNPs. An enhanced thiol pool may in this case serve as a potent antioxidant tool for these cells.

## 4. Conclusions

Plant tobacco BY-2 cell suspension culture represents one of the most effective experimental tools in the determination of toxicity and mechanisms of action of a wide range of compounds. In our work, we were focused on the effect on non-modified and modified nanoparticles on BY-2 cell suspension culture. Significant differences between the effects of NPs and MNPs were determined. However, the mechanisms of their toxicity remain almost unknown. Uptake, effect of NPs and MNPs on other metal ions uptake, compartmentation and possible interaction of NPs and MNPs surface with different biomolecules must be further investigated. Nevertheless, this work represents a useful pilot study focused on nanoparticles, which have never been investigated on plant cell models. 
